# A new approach for the analysis of bacterial microarray-based Comparative Genomic Hybridization: insights from an empirical study

**DOI:** 10.1186/1471-2164-6-78

**Published:** 2005-05-27

**Authors:** Eduardo N Taboada, Rey R Acedillo, Christian C Luebbert, Wendy A Findlay, John HE Nash

**Affiliations:** 1 Pathogen Genomics Group, Institute for Biological Sciences, National Research Council, 100 Sussex Drive, Ottawa, Ontario, K1A 0R6, Canada

## Abstract

**Background:**

Microarray-based Comparative Genomic Hybridization (M-CGH) has been used to characterize the extensive intraspecies genetic diversity found in bacteria at the whole-genome level. Although conventional microarray analytical procedures have proved adequate in handling M-CGH data, data interpretation using these methods is based on a continuous character model in which gene divergence and gene absence form a spectrum of decreasing gene conservation levels. However, whereas gene divergence may yet be accompanied by retention in gene function, gene absence invariably leads to loss of function. This distinction, if ignored, leads to a loss in the information to be gained from M-CGH data.

We present here results from experiments in which two genome-sequenced strains of *C. jejuni *were compared against each other using M-CGH. Because the gene content of both strains was known *a priori*, we were able to closely examine the effects of sequence divergence and gene absence on M-CGH data in order to define analytical parameters for M-CGH data interpretation. This would facilitate the examination of the relative effects of sequence divergence or gene absence in comparative genomics analyses of multiple strains of any species for which genome sequence data and a DNA microarray are available.

**Results:**

As a first step towards improving the analysis of M-CGH data, we estimated the degree of experimental error in a series of experiments in which identical samples were compared against each other by M-CGH. This variance estimate was used to validate a Log Ratio-based methodology for identification of outliers in M-CGH data. We compared two genome strains by M-CGH to examine the effect of probe/target identity on the Log Ratios of signal intensities using prior knowledge of gene divergence and gene absence to establish Log Ratio thresholds for the identification of absent and conserved genes.

**Conclusion:**

The results from this empirical study validate the Log Ratio thresholds that have been used in other studies to establish gene divergence/absence. Moreover, the analytical framework presented here enhances the information content derived from M-CGH data by shifting the focus from divergent/absent gene detection to accurate detection of conserved and absent genes. This approach closely aligns the technical limitations of M-CGH analysis with practical limitations on the biological interpretation of comparative genomics data.

## Background

Comparison of intraspecies multi-strain bacterial genome sequence data has shown that, even over short evolutionary time scales, genome evolution is dominated by gene insertions/deletions and gene divergence [1-4]. Genome levels of intraspecies genetic diversity must be examined if we are to gain a better understanding of genome evolution [5] and if we are to maximize the practical use of bacterial genome sequence information, for instance for development of technical applications, e.g., vaccine or drug development.

One of the aims of bacterial intraspecies comparative genomics is to determine the overall genetic similarity between strains. Where sequence information is available, this type of analysis relies heavily on sequence homology and centres on the determination of conserved genes, strain-specific (i.e. unique) genes and, where the sequence provides unambiguous evidence, determination of orthologous and paralogous genes [6-9]. Although it has become increasingly apparent that obtaining the sequence of multiple strains per species is highly desirable, currently these types of datasets are limited in number. In their absence, other methods for performing comparative genomics have been developed. Among them, microarray-based comparative genomic hybridization (M-CGH) based on genome-sequenced strains has shown enormous potential [10-12].

Two different microarray-based approaches have been used to study the genetic composition of unknown bacterial strains. In the first approach, a control genome-sequenced strain was used as a reference to generate the probes for a microarray [13-16]. In the second approach, microarray probes were derived from the tester strain, either from a tester-derived shotgun library or a library enriched for tester-specific DNAs [17]. With either approach, control- and tester-derived targets are co-hybridized to the microarray and control- and tester-derived signals are compared, often by computing the Log Ratio (LR) = log_2_(tester signal/control signal). Whereas genes with similar signal in either channel are expected to have LRs near zero, genes with LRs that deviate significantly from LR = 0 are likely to show copy number changes or sequence divergence between control and tester strains.

The relatively small number of studies on bacterial M-CGH has demonstrated the power of the method in a comparative genomics context despite a lack of consensus in current methods for analyzing M-CGH data. Although potential methods for standardizing and improving analysis have been suggested [15,18] in practice, M-CGH data has routinely been analyzed by categorizing genes into two groups: genes that are likely to be conserved and genes that are likely to be divergent. One notable problem with this approach is that no attempt is made to differentiate between gene divergence and gene absence, despite the significant biological and evolutionary differences implied by these two types of events. A framework for improved analysis would require empirical data on the relationship between Log Ratio (LR) from M-CGH experiments and sequence conservation levels, however, to our knowledge no studies exist that have directly examined this question.

The availability of intraspecies genome data from two strains of *Campylobacter jejuni *[19,20], has provided us with the opportunity to examine the quantitative relationship between the LR and probe/target identity (PTI) using our *C. jejuni *microarray. This experimental design allows us to directly match microarray results to the *a priori *interpretation of gene divergence and gene absence patterns. The goal of this study is to define the analytical parameters for the accurate prediction of gene conservation levels, leading to improved interpretation of M-CGH data. We present here the results of a detailed analysis of M-CGH experiments using the two genome-sequenced strains of *C. jejuni*.

## Results and discussion

### Determination of technical variation in M-CGH experiments

**Figure 1:**
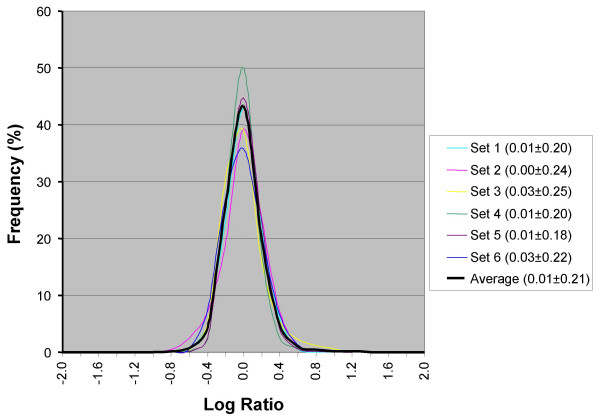
**Log Ratio distribution of self-self experiments**. The LR distribution of self-self experiments was used to determine the level of experimental variability in our experimental platform. Standardized samples of genomic DNA from *C. jejuni *NCTC 11168 (or RM1221) labelled with Cy3 and Cy5 were co-hybridized to our microarray. Results from six replicates had mean LR = 0.01 with an average SD of 0.215. Because samples from the same genomic DNA were used in both channels, LRs were expected to remain close to 0 and any deviations could be attributable to experimental error.

In order to examine the Log Ratio (LR) distributions where no differential signals are expected, we performed control experiments in which dual-labelled NCTC 11168 (or RM1221) DNA was tested in a series of self-self M-CGH experiments. Although our microarray is based on strain NCTC 11168 the resulting LRs should remain close to zero because, regardless of the strain used in the self-self experiment, "Control" and "Tester" targets are identical. Thus, any observed deviation from this result is likely due to technical variability in the assay and can be used to determine a threshold for statistically significant differential signals (i.e., outliers). The LR distribution in six replicates follows a normal distribution with a mean LR for six replicates of ~0.01 ± 0.22 (Figure 1) and, as expected, the mean and standard deviation of the various replicates were uniform regardless of the strain used. The variances observed were due to stochastic differences in the competitive hybridization of targets to the probes on the microarray and a good estimate of the technical variation in our experimental platform. Based on this data a LR = ± 1.0, used by many experimenters to identify divergent or deleted genes in similar M-CGH studies, represents a conservative threshold for divergent gene detection, since genes in which tester and control sequences are identical have a probability of less than 3.0 × 10^-6 ^of having a LR greater than 1.0

### Analysis of the Log Ratio distribution of highly conserved genes

**Figure 2:**
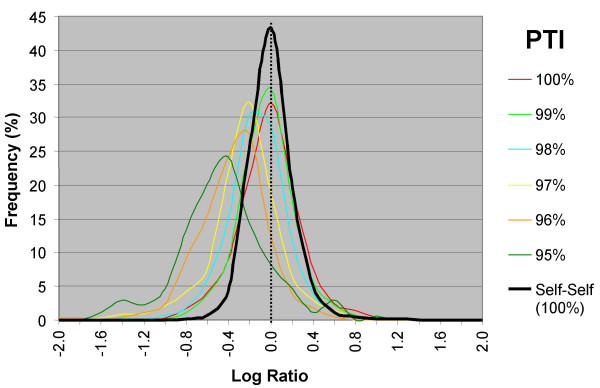
**Log Ratio distributions of highly conserved genes**. LR distributions from a series of M-CGH experiments comparing two genome-sequenced strains of *C. jejuni *(NCTC 11168 vs. RM1221). Genes were binned according to PTI and the LR distributions of bins with greater than 95% PTI are presented here. Because the microarray was designed based on strain NCTC 11168, LR deviations from 0 would be the result of sequence divergence or gene absence in strain RM1221. The LR distributions of genes with greater than 99% PTI do not deviate significantly from the average distribution of a Self-self experiment whereas increasingly larger deviations are observed in the range from 98 to 95% PTI.

We analyzed data from a set of M-CGH experiments comparing strain NCTC 11168 (Control) with strain RM1221 (Tester). Because the probes in our microarray were PCR-amplified from the Control strain, Control targets should have 100% probe/target identity (PTI) with the probes on the microarray, and the LR values observed should be a function of the PTI between Tester targets and the NCTC 11168-derived microarray probes. The LR distribution of genes with 100% identity between NCTC 11168 and RM1221 (n = 114) would be expected to behave much like that of self-self experiments because in both cases Control and Tester targets are identical and thus have 100% PTI. This was found to be the case although the distribution of genes with 100% PTI had larger standard deviation (σ = 0.28) than that of self-self experiments (σ = 0.21) (Figure 2). Genes for which the RM1221 sequence had less than 100% sequence identity with NCTC 11168 would be expected to yield LRs that deviate from 0 due to the decreased hybridization of targets that are imperfectly matched to probes on the microarray. We examined the behaviour of genes with high levels of PTI in order to determine the level of sequence divergence that would have an observable effect on LRs. We found that whereas genes with greater than 99% PTI had LR distributions that were nearly indistinguishable from those from self-self experiments, genes with as little as 2% sequence divergence (i.e., 98% PTI and below) deviated from the LR distribution of genes with 100% PTI.

### Analysis of the relationship between PTI and Log Ratio

**Figure 3:**
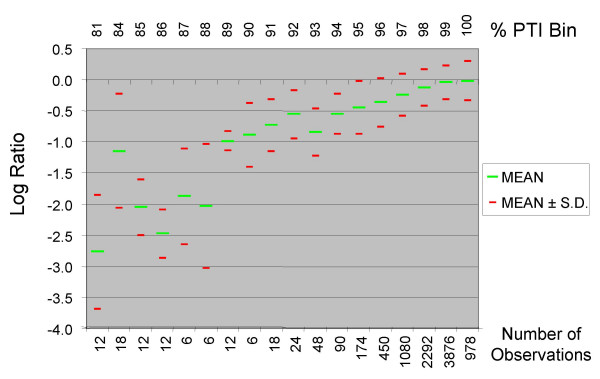
**Relationship between Log Ratio and PTI**. We plotted the average LR of genes with varying levels of % PTI from M-CGH experiments comparing two genome-sequenced strains of *C. jejuni *(NCTC 11168 vs. RM1221). Genes were binned according to PTI and the LR distributions of individual bins are presented here. The number of observations made within each PTI bin is shown in the lower axis. As seen in Figure 2, the average LR of genes with high levels of PTI are very similar, although a noticeable decrease in average LR is observed even at 98% PTI. Although the average LR becomes increasingly negative as PTI levels drop, given the SD observed within each group PTI category, there is considerable overlap between categories.

In order to examine the relationship between % PTI and LR in greater detail, we plotted the mean LR of genes according to their % PTI (Figure 3). As shown previously, the LR distribution of genes with greater than 99% PTI were similar. However, in lower PTI ranges a small yet noticeable decrease in average LR was observed. Although the small number of genes with less than 93% identity makes it difficult to obtain meaningful LR trends because of high variance, decreasing PTI still led to increasingly negative LRs. One caveat of these observations is that the LR of individual data points within a given PTI range show sufficient variability to make PTI predictions based on LR values potentially inaccurate across most of the range of PTIs. For example, although the difference in mean LR of genes with 95% PTI and 96% PTI is 0.12, their standard deviations are 0.42 and 0.39, respectively. Thus, although the average LR decreased with decreasing PTI, there is considerable overlap between the distributions.

### Analysis of the Log Ratio distribution of absent genes

**Figure 4:**
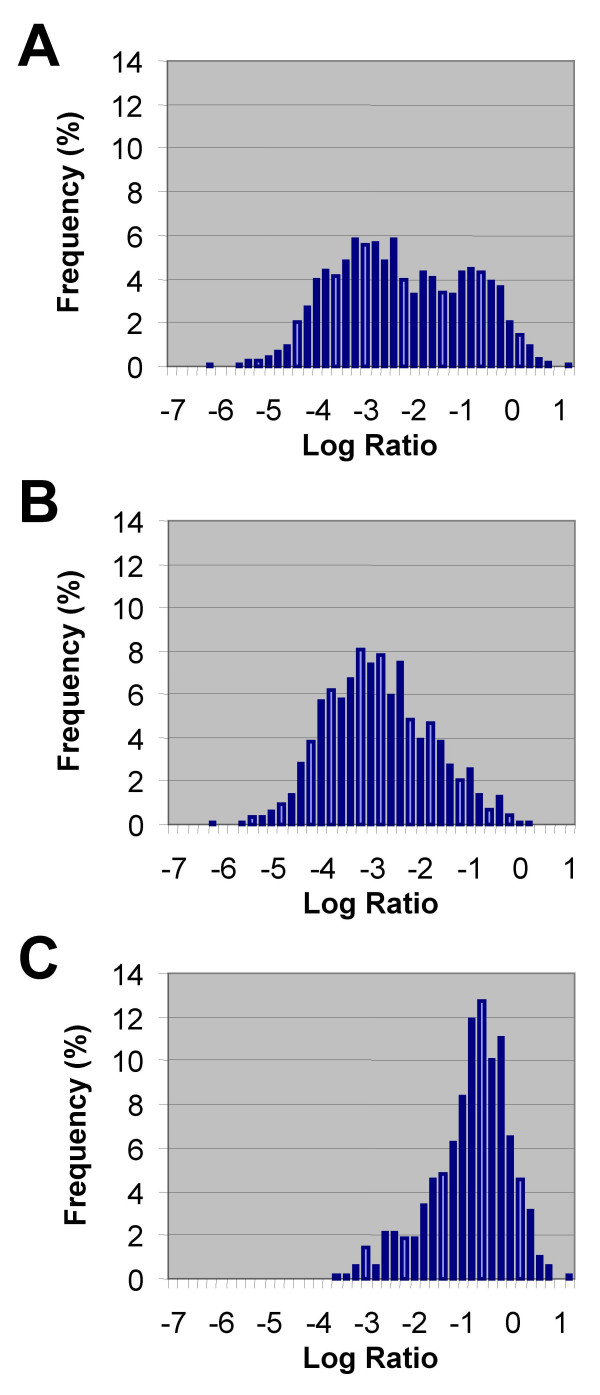
**Log Ratio distribution of genes absent from strain RM1221**. We plotted the LR distribution of genes predicted to be absent from strain RM1221 based on BLAST searches of NCTC 11168 against the RM1221 genome. Because of the lack of Tester signal predicted from these genes, LRs should be expected to be highly negative. The LR distribution (A) appeared to be bimodal with a significant number of genes bearing unusually high LRs. Further examination of these genes revealed a potential bias towards genes represented by short probes on the microarray (i.e. less than 250 bp). Separate re-plotting of the LR distributions of long (B) and short (C) probes confirms a higher average LR among genes with short probes.

Genes that are absent in RM1221 (ie. 0% PTI) should have highly negative LRs because they should yield detectable hybridization signal in the Control channel coupled with a lack of signal in the Tester channel. Although the LR distribution of genes with 0% PTI was shifted towards the left (Mean = -2.34 ± 1.35), it also appeared to be bimodal, with a number of genes with higher than expected LR. When these genes were examined more closely, a common feature was a short microarray probe size (< 250 bp). We plotted the LR distribution of genes with probe sizes <250 bp and >250 bp separately, and found a significant difference in their respective LR distributions (Figure 4). Whereas the LR distribution of the former was -0.93 ± 0.82, the LR distribution for longer genes was -2.94 ± 1.04. This "dampening" in LR amplitude appears to be largely the effect of an overall diminished signal for short genes (results not shown), possibly due to a difference in hybridization kinetics or hybrid stability under the hybridization and washing conditions used. The effect of decreased signal is that of decreased dynamic range because a lower signal in the control channel restricts the amplitude of the LR that can be measured.

### Determination of thresholds for highly conserved and absent genes

**Figure 5:**
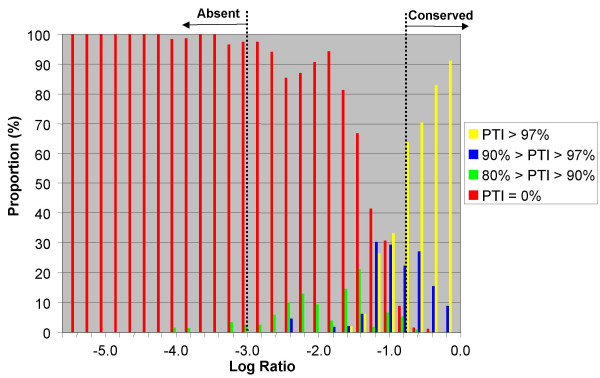
**Determination of thresholds for absent and conserved genes**. We calculated the proportion of genes belonging to each of four PTI categories at 0.2 LR intervals in order to determine LR thresholds that could be used to predict absent and conserved genes with a high degree of certainty. Below a LR of -3.0, the false positive rate for conserved genes is less than 1%; similarly, the false positive rate for absent genes above LRs of -0.8 is also less than 1%. In the LR interval between -3.0 and -0.8, particularly approaching the -0.8 boundary, there are significant number of genes from more than one PTI category and thus there is significant risk of misclassification.

One of our goals for this analysis was to determine whether the observed trends would enable us to predict the PTI in M-CGH experiments based on LR alone. Although the levels of technical variability mask the subtle effect that low levels of sequence divergence have on LR, the LR distributions at the two PTI extremes (>98% and 0%), which correspond to highly conserved and absent genes, show very little overlap. This enabled us to establish thresholds that, with high confidence, can be used to predict absent and highly conserved genes in M-CGH data (Figure 5). After removal of genes with short amplicon-based probes from our analysis, we established that less than 1% of the observed LRs > -0.8 originated from absent genes (31 of n = 7910). Similarly, less than 1% of observed LRs < -3.0 originated from conserved genes (6 of n = 636) with all false-positive observations stemming from the *pyrC *gene, which has a PTI of only 81.2%. Of 808 observed LR measurements in the range between -3.0 and -0.8, only 221 (27.3%) originated from genes with greater than 90% PTI. Although, based on our empirical data, LR values that fall between these two thresholds are likely to be from either absent or significantly divergent genes and unlikely to be from highly conserved genes, there is significant overlap between LR distributions of absent and divergent genes. At LRs ≅ -1.4, an observation has a nearly equal likelihood of stemming from an absent gene as it does from a present gene and thus the two classes cannot be distinguished in this LR range.

## Conclusion

Microarray analysis, whether in the context of gene expression or M-CGH studies, is based on determining which genes have statistically significant differential hybridization signal between two samples. In M-CGH analysis, these differential signals are the result of sequence divergence or differences in copy number. Two critical issues rise to the forefront in M-CGH analysis: a) does a gene show genuine differential signal (i.e. outside the norms of variability due to experimental error); b) what is the nature of the event that gave rise to the differential signal (i.e. sequence divergence, copy number change)?

Because M-CGH generates hybridization data as a proxy for sequence similarity data, it is important that it be analyzed as such. While some empirical work has been carried out on probe/target identity (PTI) and data analysis using the microarray platform, the focus has largely been on optimization of species detection and/or identification in complex samples [21-23]. In these applications, the primary goal is that of optimizing probe sets and hybridization conditions to maximize the specificity of species-specific probe/target interactions, possibly at the expense of decreased assay sensitivity and thus the majority of microarrays used for species identification are oligonucleotide-based. By contrast, in comparative genomics, the primary goal is that of gene detection for the purpose of characterizing gene content, and thus the focus must shift to detection sensitivity in order to minimize the likelihood of false positive calls on gene absence events. Because oligonucleotide-based arrays can lead to erroneous gene absence calls [24], the majority of M-CGH studies have used amplicon-based microarrays, which are more sensitive albeit at the expense of specificity [25].

A common thread among bacterial M-CGH studies has been the grouping of all outliers into a single category. Currently it is unclear whether divergent and absent genes can be distinguished based on LR data alone. Although the lack of distinction between these types of events does not negate the results from these studies, it can potentially restrict further analysis of the data. For example, in any pair of intraspecies genomes, sequence similarity can be used to define genes absent in one or the other strain as well as genes that are conserved in both strains. Although the "biological interpretation" in the case of gene absence is unambiguous, many possibilities arise when sequences share any level of similarity. For instance, single nucleotide substitutions can lead to truncated or inactive gene products. Additionally, the level of sequence similarity required for full functional homology varies from gene to gene, increasing the complexity of the analysis even when DNA sequences are directly available. The inexact nature of hybridization analysis further compounds the difficulty in interpreting signal from divergent genes by M-CGH, and thus focusing on conserved and divergent genes ignores the increased reliability of gene absence calls.

In previous work, we presented data suggesting that highly negative LR values were consistent with gene deletion events, paving the way for making the distinction between divergent and absent genes based on LR data [16]. When M-CGH data is analyzed such that gene absence events are grouped together with all other gene divergence events (i.e. as a continuous character model), it represents a significant loss of information both from a technical and from a biological point of view. In addition to the greater ambiguity in data interpretation as LRs approach the threshold for gene conservation, the continuous character approach negates the functional distinction that can be made between gene absence and gene divergence events. Because the LR thresholds described here could be used to reliably predict gene absence and gene conservation, it would be advantageous to focus the analysis of M-CGH data on the accurate detection of conserved and absent genes. While the data between the two thresholds should not be altogether discarded, the two thresholds represent boundaries defining regions in which gene absence and gene presence can be predicted with high confidence and thus should be given greater weight in subsequent analytical steps. It is important to note that the exact value of the LR thresholds presented here is specific to our experimental platform. The prediction accuracy achieved was remarkably high because of the uniform levels of variance across the multiple replicates analysed and because of the high correlation coefficients between replicates (the average ρ ≅ 0.92). This dataset was highly idealized because the relatively small number of replicates was carried out in such a way as to minimize technical variation. Nevertheless, a previous study in which we applied the thresholds described here on a large dataset showed that LRs below our "absence threshold" correlated very highly with other potential indicators of gene deletion [16].

Given the many documented sources of technical variability that can influence microarray results (e.g. variation in handling between individual investigators, laboratory conditions, microarray print batches), thresholds for gene presence/absence detection should be calibrated to the differential levels of technical variance found in individual microarray experiments, especially in large datasets. Kim et al [18] have suggested a solution to array-specific variance and normalization bias by determining thresholds specific to each array based on the point at which the LR distribution deviates from its inferred normal distribution. In practice, we have found that this approach can be susceptible to "narrow" LR distributions, leading to relaxed thresholds that yield an increased number of false positives for gene divergence. An alternative approach to deal with unequal variances and normalization biases across a dataset is based on normalizing multiple microarrays using the Z-score transformation [26,27], in which LR values are divided by the standard deviation of the LR data distribution. Z score-based metrics could be used to replace Log Ratio-based metrics, enabling direct comparisons that are more valid because data from each microarray is "variance-calibrated".

Based on the higher than expected Log Ratio values obtained in the case of absent genes, the "relative accuracy" of Log Ratio measurements obtained from short probes is significantly compromised under the standard hybridization conditions we used. It is important to note however, that based on the average standard deviations observed (< 250 bp = 0.68; > 250 bp = 0.85), results obtained from short probes do not lack precision compared to those obtained from longer probes. Nevertheless, our results show that data obtained from short probes yield anomalously high Log Ratio values. It is only because our assay represents a closed system in which all components are known that we were able to determine that short probes can significantly underestimate Log Ratio measurements. These results would not have been readily apparent in a typical experiment since there would be no *a priori *knowledge on expected Log Ratio values. Although these results were obtained in a series of CGH experiments, the anomalous Log Ratio data from short probes is likely to be encountered under any type of microarray hybridization experiment, including gene expression-profiling experiments. Although longer probes performed better in our assay, this is likely a result of the higher signal intensity obtained with long probes relative to short probes. Optimal hybridization and scanning conditions for long probes would likely be sub-optimal for short probes, leading to decreased signal and a concomitant drop in Log Ratio amplitudes. Thus the problem resides not in probe length *per se*, but rather in the mixed probe lengths encountered in our microarray. These results have important implications towards microarray probe design because the adverse probe-length effect could be mitigated through standardizing probe length. Failing that, it would be advantageous to incorporate probe length effects into any analytical framework.

The results presented here have been used to examine the relationship between LR and sequence conservation. The variability inherent in hybridization-based approaches makes it unlikely that LR data from M-CGH experiments can be used to accurately predict the level of sequence identity among divergent genes. In view of the considerable ambiguity in interpreting the significance of gene divergence even when sequence information is available, the focus on gene divergence in M-CGH studies must be re-assessed. We have established thresholds for the use of LR values for the accurate detection of highly conserved and absent genes, which should increase the robustness of downstream data interpretation and should extend the range of biological interpretation of M-CGH data. An accurate determination of conserved and absent genes should increase the accuracy of strain genotyping, metabolic pathway prediction, and determination of conserved targets for vaccine or drug development from M-CGH data.

## Methods

### Bacterial strains and genomic DNA isolation

Strain RM1221 was obtained from Food Safety and Health Research Unit, USDA. Strain NCTC 11168 was obtained from the American Type Culture Collection (Mannassas, VA). Genomic DNA isolation was carried out as previously described [16]

### Construction of a *C. jejuni *NCTC 11168 open reading frame DNA microarray

The DNA microarrays used in this work were previously described in [16]. Additional information can be obtained at [28].

### Microarray hybridizations

Genomic DNA was sheared to a mean fragment size of 1.5 Kb by nebulization in 35% glycerol at 15 PSI for 45 seconds as described by [29]. For each sample, 5 μg of sheared DNA were fluorescently labelled using direct chemical coupling with the Label-IT (Mirus Corp., Madison, WI) cyanine dyes Cy3 and Cy5 as recommended by the manufacturer. Probes were purified by sequentially passing samples through SigmaSpin (Sigma, Oakville, ON) and Qiaquick (Qiagen, Mississauga, ON) columns. Labelled DNA sample yields and dye incorporation efficiencies were calculated using a Nanodrop ND-1000 spectrophotometer (Nanodrop, Rockland, DE). Microarray hybridizations were set-up by co-hybridizing 2 ug of differentially labelled genomic DNA samples and were carried out as previously described [16]. NCTC 11168 versus RM1221 hybridizations were carried out in triplicate. A set of dye-swap experiments was also carried out, giving a total of 6 replicate experiments. Self-self hybridization experiments were carried out in which separate NCTC 11168 (or RM1221) genomic DNA samples were labelled with each Cy-dye and co-hybridized to the array.

### Data acquisition and analysis

Microarrays were scanned and analyzed as previously described [16]. Briefly, microarrays were scanned using a Chipreader laser scanner (BioRad, Mississauga, ON) according to the manufacturer's recommendations. Spot quantification, signal normalization and data visualization were performed using the program ArrayPro Analyzer (version 4.5; Media Cybernetics, Silver Spring, MD). Net signal intensities were obtained by performing local-ring background subtraction and spots with a signal less than 5-times above background were excluded from the analysis. Signal intensities for replicate spots were averaged and data from each channel were adjusted by sub-array normalization using cross-channel Loess regression. The ratio of tester signal to control signal for each gene was transformed to its base 2 logarithm [30], log_2 _[Tester Signal / *C. jejuni *NCTC 11168 Signal], hereafter referred to as "Log Ratio" (LR). LRs from the two "within slide" spot replicates were averaged. To increase the number of observations for statistical purposes, LR data from each microarray replicates were analyzed separately.

### Determining level of sequence identity between probes and targets (PTI)

We used the BLAST software package [31] to determine the identity between microarray probes and predicted target sequences. Complete genome sequence information for *C. jejuni *NCTC 11168 and *C. jejuni *RM1221 was downloaded from the National Center for Biotechnology Information's Prokaryotic Genomes Database [32], GenBank records AL111168 and CP000025, respectively. We created BLAST databases from the nucleotide sequences of the open reading frames in each *C. jejuni *genome strain and queried them with the nucleotide sequences of each probe in our microarray using the BLASTN program. The percent identity of the best hit for each subject/query pair was determined.

## List of abbreviations

M-CGH: microarray-based comparative genomic hybridization; LR: Log Ratio; PTI: Probe/Target Identity; SD: standard deviation (or σ)

## Authors' contributions

ENT designed M-CGH experiments, carried out downstream data analysis, and drafted the manuscript. RRA assisted with downstream data analysis. WAF carried out BLAST analysis of the two sequenced genomes. CCL carried out hybridizations and performed upstream data analysis. JHEN participated in the conception and supervised the design of the study and writing the manuscript. All authors submitted comments on drafts and read and approved the final manuscript.
